# Intravenous Fluid Administration May Improve Post-Operative Course of Patients with Chronic Subdural Hematoma: A Retrospective Study

**DOI:** 10.1371/journal.pone.0035634

**Published:** 2012-04-20

**Authors:** Miroslaw Janowski, Przemyslaw Kunert

**Affiliations:** 1 Department of Neurosurgery, Medical University of Warsaw, Warsaw, Poland; 2 NeuroRepair Department, Mossakowski Medical Research Centre, Polish Academy of Science, Warsaw, Poland; University of California Los Angeles, United States of America

## Abstract

**Background:**

The treatment of chronic subdural hematoma (cSDH) is still charged of significant risk of hematoma recurrence. Patient-related predictors and the surgical procedures themselves have been addressed in many studies. In contrast, postoperative management has infrequently been subjected to detailed analysis. Moreover variable intravenous fluid administration (IFA) was not reported in literature till now in the context of cSDH treatment.

**Methodology/Principal Findings:**

A total of 45 patients with cSDH were operated in our department via two burr hole craniostomy within one calendar year. Downward drainage was routinely left in hematoma cavity for a one day. Independent variables selected for the analysis were related to various aspects of patient management, including IFA. Two dependent variables were chosen as measure of clinical course: the rate of hematoma recurrence (RHR) and neurological status at discharge from hospital expressed in points of Glasgow Outcome Scale (GOS). Univariate and multivariate regression analyses were performed. Hematoma recurrence with subsequent evacuation occurred in 7 (15%) patients.

Univariate regression analysis revealed that length of IFA after surgery influenced both dependent variables: RHR (p = 0.045) and GOS (p = 0.023). Multivariate regression performed by backward elimination method confirmed that IFA is a sole independent factor influencing RHR. Post hoc dichotomous division of patients revealed that those receiving at least 2000 ml/day over 3 day period revealed lower RHR than the group with less intensive IFA. (p = 0.031).

**Conclusions/Significance:**

IFA has been found to be a sole factor influencing both: RHR and GOS. Based on those results we may recommend administration of at least 2000 ml per 3 days post-operatively to decrease the risk of hematoma recurrence.

## Introduction

Nowadays the diagnosis of chronic subdural hematoma (cSDH), in general, conveys favourable prognosis [1]. It results from relatively high success rate of surgical treatment. Despite that cSDH evacuation is charged of significant risk of hematoma recurrence [2]. Thus the treatment of cSDH still attracts attention of clinical researchers. It was evinced by controlled randomized trial published recently in Lancet [3]. The benefit of drain placement following cSDH evacuation has been proven by this study. In our department, like in North America [4], such practice was established long ago without previous evidence-based data.

Independent predictors of cSDH recurrence have been identified in several articles. However they mostly consist of non-modifiable factors such as hematoma characteristics and co-morbidities [5], [6], [7], [8]. They are valuable for counselling patients and their families but are of less importance for the modification of treatment process. Other set of articles recently pointed at the two burr hole craniostomy as a procedure of choice for cSDH evacuation, by this way authorizing surgical approach used in our department for years [9], [10], [11], [12], [13], [14].

In contrast to patient-related factors and the surgical procedure itself, the management of patients following surgery for cSDH has been infrequently subjected to detailed analysis. However it can be an important factor warranting further progress in improvement of treatment results. Since, to the best of our knowledge, the influence of intensity of intravenous fluid administration during post-operative period on clinical course of patients operated due to cSDH was not evaluated till now, we included it into analysis.

## Methods

### Objective

The evaluation of factors influencing outcomes in patients with cSDH treated surgically including those related to the post-operative care such as intravenous fluid administration (IFA).

### Participants

A total of 45 patients with cSDH were operated in our department via burr-hole craniostomy within one calendar year. Patients' age ranged from 24 to 86 (mean 66) yrs. The ratio of men to women was 2∶1. On admission patients were in good neurological condition −13–15 pts GCS. Hemiparesis did not exceed 4 pts Lovett scale. Bilateral haematomas were present in 3 (7%) patients. Abnormal coagulation profile was diagnosed in another 6 (14%) patients – they were operated only after profile correction. Hematocrit and Natrium values of all patients were within normal range (39–50% and 136–143 mmol/l, respectively).

### Description of procedures

Patients with cSDH were routinely operated in our department as emergency cases, without any delay. The surgeries were performed under general anaesthesia, however since it is very short, the kind of anaesthesia should not influence the fluid intake by patients in the post-operative period. Downward drainage was left in hematoma cavity for a one day. Drainage placement was abandoned in one patient only, because of intra-operative brain re-expansion.

The intravenous fluid therapy consisted of crystalloids. Fluids were infused slowly, at a rate of 125 ml/h. Between drips patients were mobilized. Beside intravenous fluid administration patients had free access to food and drink. Patients were kept in hospital until surgical wound got healed. Usually it was 7 days following surgery and 10 days after re-operation. Head CT scan and evaluation of neurological status were performed in all patients at discharge from hospital.

### Statistical methods

Independent variables (factors) selected for the analysis were related to various aspects of therapy (Table 1). Two dependent variables were chosen as a measure of clinical course: the rate of hematoma recurrence (RHR) and neurological status at discharge from hospital expressed in points of Glasgow Outcome Scale (GOS). Then both: independent and dependent variables were subjected to correlation analyses using Pearson correlation coefficients (PROC CORR, SAS, version 9.2) to exclude redundancy.

**Table 1:** Description of independent variables selected for regression analysis.

Therapy aspect	Independent variable	Units of variable measure	Variable description
Patient demographics	Age	years	Patients' age on admission to hospital
	Gender	male vs female	Patients' gender
Patient neurological status on admission to hospital	GCS	points	Glasgow Coma Scale (GCS)
	Markwalder	points	Markwalder scale
	ND	YES vs. NOT	Presence of neurological deficit
Laboratory tests on admission to hospital	Na	mmol/l	Level of Natrium in serum
	Hct	Percents (%)	Value of Hematocrit in blood
	ACP	YES vs. NOT	Abnormal coagulation profile
Hematoma characteristic	HT	millimetres	Thickness of hematoma
	BLH	YES vs. NOT	Bilateral hematoma
Treatment characteristic	Competence	(S vs T)	The level of surgeon competence (specialist or on-training). However all surgeries were supervised by attending neurosurgeons.
	IFA	days	The number of days after surgery when fluids were administered intravenously

Univariate and multivariate regression analysis has been performed using maximum likelihood (ML) approach (PROC LOGISTIC, SAS, version 9.2). If quasi-complete separation of data points were detected, Firth penalized maximum likelihood (FPML) method has been employed. Likelihood ratio (LR) has been used for testing global null hypothesis, with significance level set at 0.05. Backward selection process during multivariate regression analysis has been performed manually as current version of SAS does still not support Firth bias correction method. Prior to backward selection initiation the results of independent variable correlation has been analysed in detail to exclude similar and highly interdependent factors disturbing the process of multivariate regression.

Significant influence of IFA on clinical course of patients has been found. It warranted supplementary analysis of this factor. The volume of ca. 2000 ml/day is considered as a daily water demand thus the number of postoperative days when fluids were administered in at least such amount has been added to the analysis and named as full fluids (FF). It enables to shape the easy to memorize for clinicians “take home message", beyond the rather difficult to apply in daily practice, results of multivariate regression analysis. Finally patients were divided into two groups with regard to FF length: one with administration of at least 2000 ml/day over at least 3 postoperative days (high-dose fluid administration, HDFA) and the other with administration of at least 2000 ml/day for a period shorter than 3 days postoperatively (low-dose fluid administration, LDFA).

### Ethical issues

Since this is retrospective analysis of conventionally treated patients, without disclosure of their identity, no ethics approval from Institutional Review Board was necessary. The amount of intravenous fluids administered to patients depended on preference of physician taking care in post-operative period. Some physicians routinely withdrew drips within 1–2 days, while others routinely kept them over several days. Such situation takes place as no medical standard regarding the post-operative fluid therapy for patients with cSDH was set yet. Informed consent for surgery was obtained from each patient. Since it is not prospective study no consent for inclusion into the study was possible to receive at all. In fact, retrospective analysis of treatment results in many neurosurgical conditions is a part of clinical duties in our department, and only outstanding findings are prepared for publication.

## Results

Hematoma recurrence with subsequent evacuation occurred in 7 (15%) patients. At discharge from hospital 42 patients experienced good recovery (5 pts GOS). Moderate disability (4 pts GOS) was observed in remained 3 patients due to residual neurological deficit which was present preoperatively. Hematoma recurrence did not worse an outcome. All patients subjected to re-operation recovered satisfactorily. No complication of general anaesthesia and severe medical consequences during postoperative period were observed.

No correlation between dependent variables has been found (phi = −0.11, p = 0.45). The interdependence of independent variables has been revealed in several cases only (Table S1). Based on that Markwalder scale has been excluded from backward multivariate regression due to high correlation with other more detailed indicators of neurological status on admission to hospital.

Univariate regression analysis revealed that length of IFA after surgery influenced both dependent variables: RHR (p = 0.045) and GOS (p = 0.023) (Table 2). The reduction of period of post-operative IFA increased the risk of hematoma recurrence and was related to worse outcome at discharge from hospital. No one of other independent variables such as: age, gender, neurological status on admission to hospital, laboratory exams, hematoma characteristic and the level of surgeon competence influenced the rate of hematoma recurrence. Patient outcome (GOS) has not also been affected by above factors except of Natrium level in serum.

**Table 2:** Univariate regression analysis.

Independent variable	Dependent variable	Odds ratio estimates			Likelihood ratio (chi-square)	P value
		Point estimate	95% Wald confidence limits			
Age	RHR	0.983	0.935	1.033	0.442	0.506
	GOS	1.112	0.950	1.303	3.110	0.078
Gender	RHR	2.100	0.399	11.066	0.742	0.389
	GOS	5.636	0.464	68.464	1.981	0.159
GCS	RHR (F)	0.849	0.227	3.175	0.061	0.805
	GOS	0.668	0.097	4.619	0.164	0.689
Markwalder	RHR	1.249	0.242	6.455	0.073	0.787
	GOS (F)	3.102	0.167	57.700	0.736	0.391
ND	RHR	0.833	0.164	4.239	0.048	0.826
	GOS (F)	0.143	0.006	3.149	2.339	0.126
Na	RHR	1.064	0.663	1.708	0.066	0.797
	GOS	2.285	0.924	5.655	4.199	**0.040**
Hct	RHR	1.124	0.916	1.380	1.232	0.267
	GOS	0.798	0.542	1.175	1.471	0.225
ACP	RHR (F)	3.001	0.121	74.356	0.676	0.411
	GOS	0.270	0.021	3.552	0.860	0.354
HT	RHR	0.982	0.884	1.091	0.118	0.732
	GOS	0.948	0.802	1.120	0.428	0.513
BLH	RHR (F)	1.478	0.044	49.592	0.068	0.794
	GOS (F)	0.620	0.017	22.595	0.080	0.777
Competence	RHR	1.458	0.249	8.537	0.181	0.670
	GOS	1.111	0.093	13.295	0.007	0.933
IFA	RHR	0.599	0.343	1.051	4.021	**0.045**
	GOS	0.358	0.118	1.093	5.168	**0.023**

Significant values are featured with bold font. Abbreviations: GCS: Glasgow Coma Scale, ND: Neurological Deficit, Na: Natrium, Hct: Hematocrit, ACP: abnormal coagulation profile, HT: Thickness of Hematoma, BLH: Bilateral Hematoma, IFA: Intravenous Fluid Administration, RHR: Rate of Hematoma Recurrence, GOS: Glasgow Outcome Scale.

Multivariate regression performed by backward elimination method confirmed that IFA is a sole independent factor influencing RHR (Table 3). GOS has been affected by a combination of 2 factors: IFA and Na (Table 4).

**Table 3:** Multivariate regression analysis: summary of backward independent variable elimination process for dependent variable RHR.

Step	Effect removed	DF	Number In	Wald Chi-square	Pr>Chisq
1	BLH	1	10	0.0009	0.9762
2	ACP	1	9	0.0024	0.9607
3	ND	1	8	0.1013	0.7503
4	Na	1	7	0.2953	0.5869
5	Age	1	6	0.2595	0.6105
6	GCS	1	5	0.6064	0.4361
7	Gender	1	4	0.4600	0.4976
8	Competence	1	3	1.5441	0.2140
9	HT	1	2	0.8610	0.3535
10	Hct	1	1	1.0966	0.2950

Effect IFA remained in model; LR = 4.021; **p = 0.0449**.

Significant values are featured with bold font. Abbreviations: GCS: Glasgow Coma Scale, ND: Neurological Deficit, Na: Natrium, Hct: Hematocrit, ACP: abnormal coagulation profile, HT: Thickness of Hematoma, BLH: Bilateral Hematoma, IFA: Intravenous Fluid Administration, RHR: Rate of Hematoma Recurrence.

**Table 4:** Multivariate regression analysis: summary of backward independent variable elimination process for dependent variable GOS.

Step	Effect removed	DF	Number In	Wald Chi-square	Pr>Chisq
1	GCS	1	10	0.0012	0.9725
2	Competence	1	9	0.0033	0.9542
3	ACP	1	8	0.0000	0.9946
4	Age	1	7	0.0109	0.9169
5	Hct	1	6	0.0186	0.8915
6	HT	1	5	0.0084	0.9270
7	BLH	1	4	0.0515	0.8204
8	Gender	1	3	0.3093	0.5781
9	ND	1	2	0.0210	0.8848

Combination of effects IFA and Na remained in model; LR = 9.7944; **p = 0.0075**.

Significant values are featured with bold font. Abbreviations: GCS: Glasgow Coma Scale, ND: Neurological Deficit, Na: Natrium, Hct: Hematocrit, ACP: abnormal coagulation profile, HT: Thickness of Hematoma, BLH: Bilateral Hematoma, IFA: Intravenous Fluid Administration, GOS: Glasgow Outcome Scale.

Supplementary analysis revealed that FF is also significant predictor of RHR (p = 0.036). Dichotomous division of patients with relation to FF revealed that HDFA group experienced lower rate of hematoma recurrence than LDFA one (p = 0.031) (Table 5).

**Table 5:** Supplementary univariate regression analysis of IFA factor.

Independent variable	Dependent variable	Odds ratio estimates			Likelihood ratio (chi-square)	P value
		Point estimate	95% Wald confidence limits			
FF	RHR	0.480	0.219	1.049	4.383	**0.036**
	GOS	0.419	0.117	1.502	2.563	0.109
HDFA vs LDFA	RHR	11.005	0.537	225.534	4.633	**0.031**
	GOS	4.358	0.194	98.073	1.235	0.266

Significant values are featured with bold font. Abbreviations: FF: Full Fluids, HDFA: High-Dose Fluid Administration, LDFA: Low-Dose Fluid Administration, RHR: Rate of Hematoma Recurrence, GOS: Glasgow Outcome Scale.

## Discussion

We report on the regression analysis of cSDH treatment. Relatively homogenous group of patients was presented in this study with regard to good neurological status on admission to hospital. It probably determined favourable outcome at discharge. Despite that the rate of hematoma recurrence was significant.

The major finding of this study was a positive influence of intravenous fluid administration during post-operative period on the clinical course of patients with cSDH. The role of appropriate hydration of patients following more complex diseases such as subarachnoid haemorrhage is well recognized [15]. However this issue was not raised in literature in the context of cSDH which is simpler entity, but appropriate fluid delivery may facilitate brain re-expansion thus avoiding hematoma recurrence and accelerating patient recovery.

As fluid therapy can not be continued endlessly the direct inference from multivariate regression analysis is rather impractical. Thus we divided patients post hoc into 2 groups with regard to intensity of fluid therapy. Based on those results we feel to be entitled to recommend administration of at least 2000 ml per 3 days post-operatively to decrease the risk of hematoma recurrence.

Additionally, in our opinion fluid therapy should be included in forthcoming investigations focused on cSDH treatment. The application of technical advances such as pulse dye densitometry for monitoring of fluid therapy may contribute to set a closer link between patient hydration status and the clinical course in this disorder [16]. Data accumulation might bring stronger argument and further refinements of fluid therapy, by this way give reasons for controlled randomized trial initiation to establish an evidence–based data to improve further the management of patients with cSDH.

Post-operative period in patients with cSDH has been infrequently addressed by clinical researchers. Only recently it was shown that bed rest in horizontal position following surgery positively influenced clinical course [17]. In other study head elevation by 30 degrees did not worse outcome and recurrence rate in comparison to above strict regime [18]. Theoretically, bed rest may be efficient by facilitation of brain re-expansion. Opposite approach emphasized the value of patient early mobilization post-operatively [19]. It decreased medical complications while not increasing the rate of hematoma recurrence. Our approach based on intensive fluid therapy not only may stimulate brain re-expansion but also enables early mobilization of patients between drips. By this way intravenous fluid administration may constitute a golden mean giving an advantage of bed rest and early mobilization at the same time.

The other finding of this study was that higher values of Natrium in serum of patients on admission to hospital was related to worse outcome at discharge from hospital. However on the basis of this study it is difficult to explain the reason for that, especially as the levels of Natrium were within normal range. Till now the results of laboratory examinations were not included into analyses of cSDH treatment and if this factor is to be considered in future investigations it may also shed more light on the significance of Natrium level for therapy of patients with cSDH.

The influence of level of surgeon competence might be embarrassing and it is rarely subjected to analysis. As burr hole craniostomy is frequently the first surgical procedure on the long route to neurosurgical mastership it is important to evaluate whether such training scheme does not harm patients. Patients included in this study were operated by 12 various physicians (5 specialists and 7 trainees). Neither influence of particular physician (data not shown) nor its level of competence (specialist vs trainee, p>0.05) on the clinical course of patients with cSDH has been shown. One published series of cSDH treatment by residents was also comparable to other major series reported in literature [20]. Thus there is no advantage of specialist hand virtuosity and patients may equally benefit from trainee craft. Obviously each surgery was supervised by an attending neurosurgeon.

### Limitations

However we are aware that conclusions drawn from this study are limited due to some weaknesses such as retrospective approach, relatively small group of patients, no information on loculations, CT-imaged density and co-morbidities etc., we believe that this work is worth to be published as new aspect of cSDH treatment has been shown.

### Conclusions

We hypothesize that post-operative care might be a source of further improvement of cSDH therapy and it requires more attention from researchers. Appropriate fluid administration and Natrium level may prevent hematoma recurrence and accelerate patient recovery.

## Supporting Information

Table S1 **Correlation analysis of independent variables.**
(DOC)
