# Supplementary material for: Intravenous Fluid Administration May Improve Post-Operative Course of Patients with Chronic Subdural Hematoma: A Retrospective Study
Source: PLoS One. 2012 Apr 20;7(4):e35634. doi: 10.1371/journal.pone.0035634 (PMC3331986; doi:10.1371/journal.pone.0035634)
Supplement: Table S1 — Correlation analysis of independent variables. (DOC) [file pone.0035634.s001.doc]

|  | Age | Gender | GCS | Markwalder | ND | Na | Hct | ACP | HT | BLH | competence | IFA |
| --- | --- | --- | --- | --- | --- | --- | --- | --- | --- | --- | --- | --- |
| Age  p value | 1.00000 | -0.15066  0.3232 | -0.24838  0.0999 | 0.42684  **0.0035** | 0.47416  **0.0010** | 0.08926  0.5598 | -0.25303  0.0935 | 0.10085  0.5098 | 0.20791  0.1705 | -0.00347  0.9820 | -0.01264  0.9343 | 0.01863  0.9033 |
| Gender  p value | -0.15066  0.3232 | 1.00000 | -0.16310  0.2844 | 0.15824  0.2992 | 0.09172  0.5490 | -0.20865  0.1690 | 0.27254  0.0701 | -0.24268  0.1082 | 0.25323  0.0933 | -0.02621  0.8643 | -0.14112  0.3551 | 0.10886  0.4766 |
| GCS  p value | -0.24838  0.0999 | -0.16310  0.2844 | 1.00000 | -0.59235  **<.0001** | -0.24526  0.1044 | -0.01791  0.9070 | 0.10284  0.5014 | -0.04056  0.7914 | 0.00572  0.9702 | 0.09197  0.5479 | 0.04260  0.7811 | -0.16071  0.2916 |
| Markwalder  p value | 0.42684  **0.0035** | 0.15824  0.2992 | -0.59235  **<.0001** | 1.00000 | 0.69007  **<.0001** | -0.07561  0.6216 | -0.04630  0.7626 | -0.04564  0.7659 | 0.33286  **0.0255** | 0.00000  1.0000 | -0.23973  0.1127 | 0.07664  0.6168 |
| ND  p value | 0.47416  **0.0010** | 0.09172  0.5490 | -0.24526  0.1044 | 0.69007  **<.0001** | 1.00000 | -0.00522  0.9729 | 0.08387  0.5839 | 0.04725  0.7579 | 0.43235  **0.0030** | 0.07143  0.6410 | -0.42185  **0.0039** | 0.02063  0.8930 |
| Na  p value | 0.08926  0.5598 | -0.20865  0.1690 | -0.01791  0.9070 | -0.07561  0.6216 | -0.00522  0.9729 | 1.00000 | -0.09452  0.5369 | 0.13804  0.3658 | -0.06300  0.6810 | 0.08348  0.5856 | -0.00363  0.9811 | 0.16317  0.2842 |
| Hct  p value | -0.25303  0.0935 | 0.27254  0.0701 | 0.10284  0.5014 | 0.04630  0.7626 | 0.08387  0.5839 | -0.09452  0.5369 | 1.00000 | -0.40575  **0.0057** | 0.30052  **0.0449** | -0.02396  0.8758 | 0.06660  0.6638 | 0.04258  0.7812 |
| ACP  p value | 0.10085  0.5098 | -0.24268  0.1082 | -0.04056  0.7914 | -0.04564  0.7659 | 0.04725  0.7579 | 0.13804  0.3658 | -0.40575  **0.0057** | 1.00000 | -0.15879  0.2975 | 0.18898  0.2138 | 0.18055  0.2353 | -0.24347  0.1070 |
| HT  p value | 0.20791  0.1705 | 0.25323  0.0933 | 0.00572  0.9702 | 0.33286  **0.0255** | 0.43235  **0.0030** | -0.06300  0.6810 | 0.30052  **0.0449** | -0.15879  0.2975 | 1.00000 | 0.29564  **0.0486** | -0.24789  0.1006 | -0.07880  0.6069 |
| BLH  p value | -0.00347  0.9820 | -0.02621  0.8643 | 0.09197  0.5479 | 0.00000  1.0000 | 0.07143  0.6410 | 0.08348  0.5856 | -0.02396  0.8758 | 0.18898  0.2138 | 0.29564  **0.0486** | 1.00000 | 0.17370  0.2538 | -0.10789  0.4805 |
| Competence  p value | -0.01264  0.9343 | -0.14112  0.3551 | 0.04260  0.7811 | -0.23973  0.1127 | -0.42185  **0.0039** | -0.00363  0.9811 | 0.06660  0.6638 | 0.18055  0.2353 | -0.24789  0.1006 | 0.17370  0.2538 | 1.00000 | -0.15929  0.2959 |
| IFA  p value | 0.01863  0.9033 | 0.10886  0.4766 | -0.16071  0.2916 | 0.07664  0.6168 | 0.02063  0.8930 | 0.16317  0.2842 | 0.04258  0.7812 | -0.24347  0.1070 | -0.07880  0.6069 | -0.10789  0.4805 | -0.15929  0.2959 | 1.00000 |

Table S1. Correlation analysis of independent variables.

Significant values are featured with bold. Abbreviations: GCS: Glasgow Coma Scale, ND: Neurological Deficit, Na: Natrium, Hct: Hematocrit, ACP: abnormal coagulation profile, HT: Thickness of Hematoma, BLH: Bilateral Hematoma, IFA: Intravenous Fluid Administration, RHR: Rate of Hematoma Recurrence, GOS: Glasgow Outcome Scale.
